# Concurrent Remission of Therapy-Related Acute Myeloid Leukemia and IgG-λ MGUS During Oral Decitabine–Cedazuridine: A Case Report

**DOI:** 10.3390/hematolrep18050066

**Published:** 2026-09-19

**Authors:** Pasquale Niscola, Carla Mazzone, Marco Giovannini, Valentina Gianfelici, Maria Ilaria Del Principe

**Affiliations:** 1 Hematology Unit, Medical Area Department, S. Eugenio Hospital (ASL Roma 2), 00144 Rome, Italy; carla.mazzone@aslroma2.it (C.M.); valentina.gianfelici@aslroma2.it (V.G.); del.principe@med.uniroma2.it (M.I.D.P.); 2 Hematology, Department of Biomedicine and Prevention, University of Tor Vergata Rome, 00133 Rome, Italy

**Keywords:** acute myeloid leukemia, monoclonal gammopathy of undetermined significance (MGUS), multiple myeloma, oral decitabine, epigenetic therapy

## Abstract

**Background and Clinical Relevance**: The association between acute myeloid leukemia (AML) and monoclonal gammopathy of undetermined significance (MGUS) is rare and likely coincidental, due to the similar epidemiology of these two blood disorders in older AML patients who are not eligible for intensive chemotherapy or aggressive treatment, as they can be excessively myelosuppressive. Such treatments include those containing venetoclax, an easy-to-use all-oral form of decitabine–cedazuridine (DEC-C), which is now available and approved for AML in addition to standard parenteral DNA methyltransferase inhibitors, which have been commonly used in routine clinical practice for about two decades in patients unsuitable for intensive chemotherapy (IC). **Case Presentation**: A 71-year-old woman developed a therapy-related acute myeloid leukemia (t-AML) seven years after the discovery of the plasma cell dyscrasia. She had carried an asymptomatic and non-progressing IgG-λ MGUS since 2019 while affected by breast cancer, which was cured with chemotherapy and radiotherapy. BM smear and trephine biopsy at the time of t-AML diagnosis showed 30% myelomonoblasts and 8% plasma cells, while SPEP showed the presence of 15 g/L of monoclonal protein. However, considering her age and other medical and personal history, the patient was found unsuitable for IC as well as venetoclax treatment because of the myelosuppressive effect of the therapy. Therefore, she received DEC-C oral monotherapy. After four DEC-C cycles, she achieved CR with hematological incomplete recovery (CRi). During DEC-C monotherapy, serum immunofixation (IF) and BM evaluations after 12 months of treatment showed gradual clearance of the IgG-λ paraprotein and BM plasma cells. Maintenance therapy with oral DEC-C is ongoing for 18 months after achieving CRi, without any signs of toxicity or infection. **Conclusions**: This report describes the first anecdotal case of t-AML responding to DEC-C with concomitant clearance of the paraprotein associated with MGUS.

## 1. Introduction

In patients with acute myeloid leukemia (AML), concomitant clonal plasma cell neoplasia, including monoclonal gammopathy of undetermined significance (MGUS) or multiple myeloma (MM), is extremely uncommon [1,2,3,4,5]. MM and AML are well known to be distinct diseases in the lymphoid and myeloid hematologic lineages, but recent molecular evidence suggests they may share a common origin; this view is consistent with ongoing research on clonal hematopoiesis and BM niches [2,5,6].

In this context, a highly altered BM microenvironment characterized by dysregulated immunity, poor antitumor immunity, and chronic inflammation may result in high circulating levels of interleukin-6 (IL-6), which may be permissive for clonal expansion of myeloblasts and plasma cells [5,6]. In addition, although the common origin of the two blood disorders remains unfounded and has not been demonstrated, epigenetic pathways common to tumoral plasma cells and AML have been reported [7,8,9,10,11]. They include global changes that promote genome instability and inhibit protective mechanisms; global hypomethylation that activates transposable elements and oncogenes, resulting in chromosomal instability; and hypermethylation of TSG promoters. The latter silences genes responsible for cell cycle regulation, checkpoints, and apoptosis. Moreover, upregulation of DNMT1 and DNMT3B, which catalyze DNA methylation, is a hallmark characteristic of this process. Histone methylation, along with PRC2 activity, affects chromatin accessibility and contributes to renewal. Upregulation of the histone demethylase KDM1A inhibits differentiation. Another common trait of these two cell types is the presence of BET proteins such as BRD4, which recognize acetylated sequences and support MYC-dependent transcription.

Interleukin-6 produced by stromal cells activates STAT3 signaling and upregulates DNMT and HDAC activity, thereby protecting tumor cells from chemotherapy-induced apoptosis [7,8,9,10,11]. DNA methyltransferase (DNMT) inhibitors, such as the hypomethylating agents (HMAs), have become the mainstay of treatment for patients with myelodysplastic syndromes and AML who are ineligible for intensive chemotherapy [12]. Although HMAs, such as azacytidine and decitabine, were administered parenterally, the availability and regulatory approval of an oral formulation of decitabine–cedazuridine (DEC-C) have revolutionized this field [13]. Indeed, with its fixed-dose oral regimen of 35 mg of decitabine and 100 mg of cedazuridine, exposure is comparable to intravenous decitabine without parenteral administration.

As such, DEC-C facilitates clinical management and improves health-related quality of life while maintaining significant epigenetic and cytoreductive efficacy [13]. This case report describes durable morphologic complete remission without complete hematologic response (CRi) and persistence of SPEP and serum immunofixation negativity for IgG-λ paraprotein in relation to MGUS, following oral DEC-C administration in an older, frail woman with AML.

## 2. Case Presentation

A 71-year-old female patient was under our management in 2019 after the discovery of an IgG-λ monoclonal paraprotein (15 g/L) during a routine laboratory workup. Her past medical history was not remarkable apart from pharmacologically well-controlled hypertension. At that time, she was asymptomatic except for bilateral hip osteoarthritis, for which she was being treated by orthopedic specialists using conservative and rehabilitative measures, such as local electromedical applications and physiotherapy. BM aspiration and trephine biopsy allowed for a diagnosis of MGUS. There were no signs of CRAB criteria. Test results demonstrated normal values for blood and kidney calcium levels; hemoglobin was within the normative range; the free light chain ratio was normal; urine test did not reveal any proteins or light chains; bone X-ray did not show any damage. Consequently, no treatment and/or intervention was required.

In July 2022, she had HR+ (hormone receptor positive) breast cancer; therefore, surgery and CMF (Cyclophosphamide, Methotrexate, and Fluorouracil) chemotherapy for six cycles, radiation therapy, and tamoxifen therapy were required for five years. No changes in paraprotein concentration before and after breast cancer treatment (approximately 1.4 g/L) were observed. Regular examinations of the breast combined with imaging (mammography, ultrasonography, and breast magnetic resonance imaging) revealed complete remission of breast cancer.

Therefore, the patient remained healthy and physically active until December 2025, when she complained of fatigue and dyspnea. At admission, the patient was pale and tired and required a red blood cell transfusion, as she had anemia. Her hematology workup comprised bone marrow aspiration for morphology, immunophenotyping, cytogenetics, and trephine biopsy. Her karyotype was 46, XX [20]. FISH revealed no abnormalities. Morphological analysis of BM smears showed a 30% population of medium-sized blasts positive for CD117, CD34, MPO, CD33, and CD13. In addition, BM revealed trilineage cytomorphological dysplasia and 5% of plasma cells positive for CD56 and CD138.

Considering cytotoxic exposure for breast cancer treatment, the patient was diagnosed with therapy-related AML (t-AML). Moreover, SPEP and serum immunofixation showed a definite sharp monoclonal IgG-λ paraprotein spike of about 15 g/L, without any CRAB criteria. As far as the patient’s clinical assessment was concerned, the patient was not eligible for intensive chemotherapy and only eligible for a very myelotoxic treatment using venetoclax. In other words, the patient was not fit and was frail (ECOG performance status = 2). Additionally, the patient’s body mass index was 38, and the patient had problems moving herself because she had very painful bilateral osteoarthritis. A decision was taken after discussing the matter with the patient and her family [12]. DEC-C treatment regimen was selected as it consists only of oral drugs [13]. Therefore, she was started on DEC-C orally each day from 1 to 5 within a 28-day cycle, without any break or dose reduction. DEC-C enabled the CRi of AML. After four cycles of DEC-C, BM aspiration demonstrated a CRi, with BM blasts reduced from 30% to less than 5%.

Given the patient’s condition and the lack of alternative applicable treatments, we did not assess for measurable residual disease.

The approach we used to manage this patient is consistent with our clinical practice in treating older, frail AML patients in a real-world setting. Moreover, this patient would not be included in any other trial, even in the case of non-response or disease progression. Indeed, DEC-C was intended for disease control and transfusion independence only because no other objective could be set. CRi state was preserved for 12 and 18 months. At the same time, we observed a steady decline in paraprotein levels, eventually reaching undetectability by conventional laboratory methods, such as SPEP/serum immunofixation (Table 1).

Considering the stability of MGUS since its first detection in 2019, with its features remaining unchanged before and after breast cancer treatment (MCF regimen + RT), and how the patient’s medications were limited to antihypertensive drugs and to NSAIDs in some instances, we ascribed the reduction in paraprotein levels to undetectability by conventional laboratory methods to DEC-C, in line with another reported case observed in a patient who received parenteral decitabine [14].

Notably, the monoclonal spike on SPEP and IF analyses progressively decreased over the treatment course. After 12 months of uninterrupted oral DEC-C, the monoclonal spike was negative on both SPEP and IF, confirmed by controls at 3-month intervals at 15 and 18 months. On further bone marrow evaluation, there were no plasma cells present.

This finding was replicated with oral DEC-C therapy, without hematologic side effects or infection. Detailed evaluation was not considered necessary or appropriate for the patient. Currently, 7 years and 18 months have passed since the diagnosis of MGUS and AML, respectively, and the patient remains healthy and is still on DEC-C therapy.

## 3. Discussion

A concomitant presence of AML and plasma cell dyscrasia, from MGUS to smoldering or symptomatic multiple myeloma, is a rather rare clinical scenario [2]. Cohort and registry studies consistently demonstrate that the presence of existing plasma cell dyscrasia predisposes a person to myeloid malignancies such as MDS and AML regardless of any previous exposure to treatment with DNA-damaging chemotherapy [15]. Monotherapy with DNA hypomethylating agents (mainly azacitidine and decitabine) used to treat unfit AML patients has been reported to induce dual-lineage response and paraprotein disappearance [14,16,17]. Monotherapy with decitabine in a conventional AML induction schedule (20 mg/m^2^ per day for 5 days every 4 weeks) achieved a prolonged complete remission in an older, frail patient with secondary oligoblastic AML concurrent with IgG kappa MGUS, with an initial paraprotein concentration of 15 g/L and 10% plasma cells in BM [14]. After 4 cycles of decitabine, the bone marrow blasts fully cleared and did not return even after 12 cycles. Also, a series of serum protein electrophoresis indicated a progressive disappearance of the monoclonal protein peak to levels where it could not be detected by immunofixation electrophoresis [14].

These data agree with previous reports on the complete resolution of IgG-kappa paraprotein and underlying MGUS following the treatment of chronic myelomonocytic leukemia with azacitidine [16]. Similarly, azacitidine (75 mg/m^2^ daily for 7 days every 28 days) showed a considerable antimyeloma effect in AML patients with plasma cell disorders [17,18].

Moreover, Oka et al. reported another case of AML secondary to smoldering multiple myeloma with IgG paraproteinemia (65 g/L), p53 and CD34 antigen expression, and elevated IL-6 (37 pg/mL). Azacitidine in two cycles resulted in full hematological remission of AML (blast number was reduced to 1%), BM plasma cells were reduced from 10% to 5%, IgG paraproteinemia in the serum was normalized (from 65 to 18 g/L), and circulating IL-6 was completely normalized in both cases [19].

Paraproteinemia resolved simultaneously with leukemic remission without any specific cytotoxic regimen for plasma cells [19]. The resolution of paraprotein in patients with AML treated with epigenetic therapy is associated with common molecular characteristics of myeloid and plasma cell disorders [2,7,8,9,10,11]. Hypermethylation of tumor suppressor genes occurs during both AML oncogenesis and clonal plasma cell proliferation. In vitro and clinical evidence indicates that azacitidine and decitabine cause the proteolytic destruction of DNA methyltransferases incorporated in nucleic acids.

The anti-demethylating action of HMA disrupts not only the demethylation pathway but also cytokine signaling in cells [20]. For instance, azacitidine blocks IL-6, IL-6 receptor alpha, and NF-κB pathways and suppresses Bcl-XL. In addition, reducing the number of dominant myeloid blasts reduces local bone marrow and immune dysfunction that prevents paraprotein formation. Paraprotein resolution during HMA therapy demonstrates the intrinsic anti-plasma cell effect of azacitidine and decitabine, in addition to their proven activity in managing AML [14,17,18]. Although paraprotein clearance under HMAs administered for AML has been anecdotally reported, we report the first description of this occurrence in a patient receiving DEC-C.

Indeed, despite significant diagnostic limitations in the case of a patient with a stable IgG-λ MGUS developing six years after a t-AML that was managed in daily clinical practice, our anecdotal case reports the simultaneous clearance of two cytologically distinct malignant clones under oral DEC-C monotherapy, suggesting a possible shared epigenetic and transcriptional pathway [5,6]. Notably, the CRi of AML, along with the apparent undetectability of paraprotein, was attained and sustained with oral DEC-C maintenance therapy without hematologic toxicity or infections.

## 4. Conclusions

In conclusion, the rarity of the association between AML and MGUS supports a cautious biological hypothesis, as a shared clonal origin has not yet been proven. In general, it is regarded as an incidental finding and is usually found in case reports such as the one described here. In addition, continuous disappearance below detectable levels using conventional laboratory methods has been observed in patients with AML and associated MGUS receiving parenteral azacitidine and decitabine [14,17,18].

However, to the best of our knowledge, this is the first description of such a case in patients who received DEC-C. In fact, the concurrent clearance of BM blast cells and total eradication of the monoclonal paraprotein IgG-λ, although accidental, may hint at the wide-reaching influence of epigenetic therapy through DNMT inhibition [1,2,18,19,20]. In a rapidly changing therapeutic landscape, from personalized to oral therapies, DEC-C combined with venetoclax emerges as a highly promising synergistic approach [13].

## Figures and Tables

**Table 1:** Patient’s outcome.

Treatment Timeline	Myeloid Blast Burden in BM	Plasma Cell Infiltration of BM	SPEP and IF: IgG-λ Monoclonal Band	Clinical Symptoms and Transfusion Status
Baseline (Month 0)	30%	8%	Sharp peak (15 g/L). IF: IgG-λ	Severe fatigue, pancytopenia, transfusion-dependent
Month 4 (Cycle 4)	<5% (CRi)	3%	Moderate reduction in peak concentration	Complete resolution of cytopenias, transfusion-independent
Month 12 (Cycle 12)	<5% (Confirmed CRi)	0%	Undetectable on SPEP and IF	Asymptomatic, baseline performance status restored
Month 18 (Cycle 18)	<5% (Persistent CRi)	0%	Persistently undetectable on SPEP and IF	Well, active, and asymptomatic

BM: bone marrow; SPEP: serum protein electrophoresis; IF: immunofixation; CRi: complete remission without hematological recovery.

## Data Availability

The data presented in this study are available from the corresponding author upon reasonable request. Due to privacy and confidentiality considerations related to patient information, the underlying clinical data cannot be publicly shared.
